# Wound Trauma Exacerbates Acute, but not Delayed, Effects of Radiation in Rats: Mitigation by Lisinopril

**DOI:** 10.3390/ijms21113908

**Published:** 2020-05-30

**Authors:** Meetha Medhora, Tracy Gasperetti, Ashley Schamerhorn, Feng Gao, Jayashree Narayanan, Zelmira Lazarova, Elizabeth R. Jacobs, Sergey Tarima, Brian L. Fish

**Affiliations:** 1 Department of Radiation Oncology, Medical College of Wisconsin, Milwaukee, WI 53226, USA; tgasperetti@mcw.edu (T.G.); fgao@mcw.edu (F.G.); jnarayan@mcw.edu (J.N.); bfish@mcw.edu (B.L.F.); 2 Department of Physiology, Medical College of Wisconsin, Milwaukee, WI 53226, USA; ejacobs@mcw.edu; 3 Department of Pulmonary Medicine, Medical College of Wisconsin, Milwaukee, WI 53226, USA; 4 Cardiovascular Center, Medical College of Wisconsin, Milwaukee, WI 53226, USA; 5 Research Service, Department of Veterans Affairs, Zablocki VAMC, Milwaukee, WI 53295, USA; 6 Department of Plastic Surgery, Medical College of Wisconsin, Milwaukee, WI 53226, USA; aschamerhorn@mcw.edu; 7 Department of Dermatology, Medical College of Wisconsin, Milwaukee, WI 53226, USA; lazarovazelmira@gmail.com; 8 Institute for Health and Equity, Medical College of Wisconsin, Milwaukee, WI 53226, USA; starima@mcw.edu

**Keywords:** leg-out partial body irradiation (PBI), mitigation, rat model of irradiation, combined injury

## Abstract

The goal of this study is to understand and mitigate the effects of wounds on acute radiation syndrome (ARS) and delayed effects of acute radiation exposure (DEARE), for preparedness against a radiological attack or accident. Combined injuries from concomitant trauma and radiation are likely in these scenarios. Either exacerbation or mitigation of radiation damage by wound trauma has been previously reported in preclinical studies. Female WAG/RijCmcr rats received 13 Gy X-rays, with partial-body shielding of one leg. Within 2 h, irradiated rats and non-irradiated controls were given full-thickness skin wounds with or without lisinopril, started orally 7 days after irradiation. Morbidity, skin wound area, breathing interval and blood urea nitrogen were measured up to 160 days post-irradiation to independently evaluate wound trauma and DEARE. Wounding exacerbated morbidity in irradiated rats between 5 and 14 days post-irradiation (during the ARS phase), and irradiation delayed wound healing. Wounding did not alter delayed morbidities from radiation pneumonitis or nephropathy after 30 days post-irradiation. Lisinopril did not mitigate wound healing, but markedly decreased morbidity during DEARE from 31 through 160 days. The results derived from this unique model of combined injuries suggest different molecular mechanisms of injury and healing of ARS and DEARE after radiation exposure.

## 1. Introduction

The increasing threats of radiological terrorism and nuclear accidents worldwide have resulted in dynamic national programs in the US aimed at pursuing development of medical countermeasures for radiation injuries. The National Institute of Allergy and Infectious Diseases (NIAID) promotes discoveries of relevant animal models to study the mechanisms of radiation-induced injuries as well as specific countermeasures to mitigate this damage. Ionizing radiation alone can result in a broad spectrum of biological tissue damage and lethality in mammals. Sequential injuries to multiple organs occur after exposure of whole animals to radiation, a pattern that has also been observed in humans [1]. Acute radiation syndrome (ARS) occurs first with gastrointestinal (GI) injury starting within a week after exposure followed by bone marrow toxicity in rats and humans [1]. Survivors of ARS proceed to develop the Delayed Effects of Acute Radiation Exposure (DEARE), which manifest as different sequelae such as lung (radiation pneumonitis between 42 and 90 days) and kidney (radiation nephropathy after > 90 days) injuries in rats, that vary depending on the initial dose of radiation. Other organs such as the brain, heart, etc., also manifest DEARE, but these injuries are typically only lethal after higher doses of radiation than those that cause lethal pneumonitis or nephropathy [2,3]. In a nuclear attack to a densely populated urban area, exposure to radiation will be combined with blast injuries causing skin wounds and/or burns. To date, there are limited studies of combined radiation and wound/burn injuries. In addition, there are mixed results from past studies, making it imperative to conduct further research. Some of these studies (briefly summarized below) highlight the limited knowledge of the mechanism of injury as well as its mitigation.

Most combined injuries in preclinical models have included exposure to radiation with skin wounds (thermal burns and/or incisions) or bacterial infection. Studies have been conducted in dogs, pigs, rats, guinea pigs and mice [4,5]. Combined insults frequently, but not always, result in more severe tissue damage and lethality than radiation exposure alone [5,6,7,8]. Studies have also shown improvement in radiation lethality by skin injury in mice and rats [9,10,11,12,13,14], often based on the timing of the injury relative to the radiation exposure. 

While there are several models measuring combined radiation and skin injury during ARS, there are fewer models measuring the effect of combined injury on delayed outcome (DEARE). Using a rat model, Gao et al. [12] demonstrated enhanced survival from pneumonitis induced by radiation combined with non-overlapping soft X-ray irradiation to the skin, as compared to irradiation alone. Rats were treated with 12.5 to 13 Gy X-rays to the whole thorax followed by 30 Gy non-lethal, soft X-rays to the skin (10% body surface area (BSA)). Radiation pneumonitis occurred after the peak of radiation- dermatitis had resolved. The results showed that radiation to the skin, given 3 h after whole thorax lung irradiation (WTLI), mitigated lung morbidity (radiation pneumonitis). Interestingly, the angiotensin converting enzyme inhibitor captopril accelerated the rate of wound healing in this combined injury model [12]. 

The current study was done with the same strain of rats as that used by Gao et al. [12], but with a more advanced model of combined radiation and skin injury. This new and more sophisticated model was specifically designed for relevance to a radiological terrorism scenario. Partial body irradiation (PBI) shielding part of one hind leg (leg-out PBI) was used in order to expose most of the body to ionizing radiation. Lethal GI damage, characteristic of this model, was mitigated by supportive care [15]. A minimal volume of bone marrow (~ 8% which received only 2 Gy) was spared to mitigate hematopoietic death. This allowed adequate numbers of rats to survive ARS making it possible to study DEARE [15]. The approach provides a more ‘real-world’ scenario to evaluate DEARE than isolated organ irradiations, which do not support interactions from multiple injured organs that will occur in a radiological attack or nuclear accident. This model is the only one currently available to test the effects of combined injuries on ARS and DEARE in the same animals. Additionally, the selected strain of rat demonstrates two relevant sequelae of DEARE in a short time period, which enables, for the first time, examination of radiation nephropathy in combination with full-thickness skin wounds. WAG/RijCmcr rats develop lethal pulmonary and renal dysfunction, as compared to mouse or nonhuman primate models. 

For combined injury in the rat model, skin wounds were generated by full-thickness punch biopsies, to mimic skin puncture that may occur from the blast generated in a radiological incident. Using this model along with matched controls of radiation only or skin wound only groups, the absence of morbidity (a term used here instead of ‘survival’) was the primary endpoint of the study. Morbid animals were euthanized to avoid pain and distress. Secondary endpoints for pulmonary and renal function to monitor DEARE, and wound healing to determine the effect of radiation on wound trauma, were also tracked. In summary, the current study tested the effects of radiation and trauma in a novel model of combined injury, which evaluated the ARS and DEARE continuously in the same wounded animals, to mimic a radiological incident. In addition, it tested the efficacy of the DEARE mitigator, lisinopril [15], after combined injury. 

## 2. Results

### 2.1. Combined Injury Exacerbates ARS 

**Figure 1:**
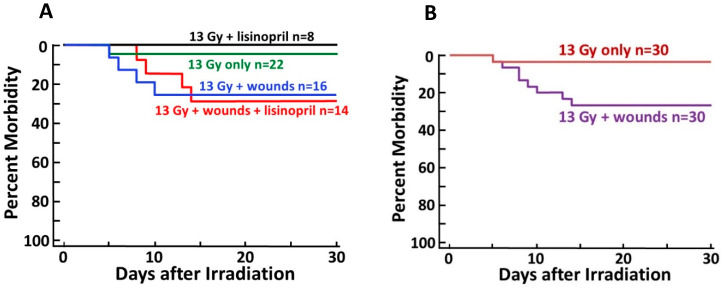
Kaplan–Meier plots depicting morbidity through 30 days post 13 Gy leg-out partial body irradiation (PBI). (**A**) All irradiated groups with or without lisinopril and/or wound trauma. Lisinopril did not significantly change 30-day morbidity. (**B**) Combined irradiated only groups (with or without lisinopril) vs. corresponding groups with wound trauma. Wounds exacerbated morbidity (*p* = 0.03) as compared to irradiated rats without wounds.

The study included six groups of rats: (1) wounds only, (2) wounds only + lisinopril, (3) radiation only, (4) radiation only + lisinopril, (5) radiation + wounds (combined injury) and (6) radiation + wounds (combined injury) + lisinopril. The drug was started 7 days after 13 Gy leg-out PBI. Since lisinopril is dissolved in drinking water, no vehicle was necessary in groups 1, 3 and 5. The rats in groups 1 and 2 were sham-irradiated. No rats were morbid in the wounds only or wounds only + lisinopril (groups 1 and 2) through ~160 days, and so they were not included in the studies for ARS that are presented in Figure 1. This figure shows a Kaplan–Meier plot for morbidity among irradiated rats in the first 30 days (during the ARS phase). There was an increase in ARS injury in the rats given combined injuries. Injuries before 7 days were considered to be due to damage to the gastrointestinal tract, and morbidity between 8 and 14 days represented damage to the bone marrow. No lethal injuries occurred between 15 and 30 days. 

The 30-day morbidity of dual injured rats with and without lisinopril was not different (odds ratio of 1.2, 95% CI of 0.17–8.26, Figure 1A), indicating lisinopril did not alter the 30-day outcome during ARS. Evaluation of the effect of lisinopril was confounded by the facts that radiation alone induced only limited lethal ARS (1/30, green line in Figure 1A and brown line in Figure 1B), and lisinopril was started 7 days after radiation when some events of lethal ARS due to GI injury had already occurred. There was a statistically significant adverse impact of wounding on 30-day morbidity (*N* = 60, *p* = 0.03 with an odds ratio of 10.2, 95% confidence interval (CI) of 1.2–482.4; Figure 1B). Wounding increased the chance of morbidity among irradiated rats by > 10-fold (Figure 1B).

### 2.2. Radiation Delays Skin Wound Healing during ARS, Which Is Not Mitigated by Lisinopril

**Figure 2:**
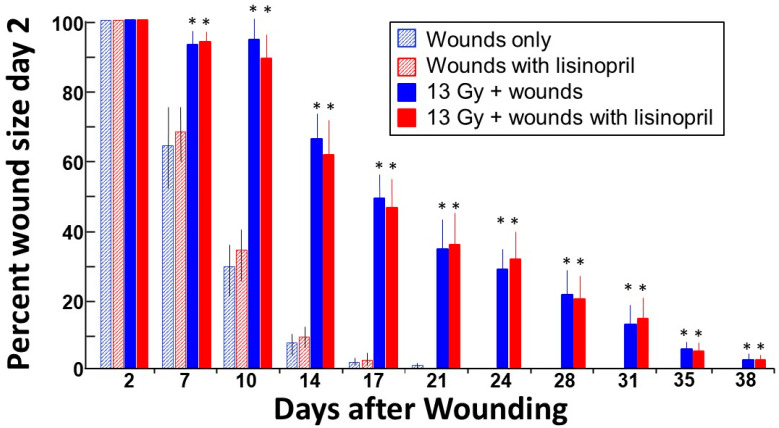
Time course of skin wound healing. All rats received skin wounds on their back as described in Materials and Methods. Wound size was measured on day 2 and fixed to 100% (Y-axis). The percent of this area that remained wounded at different time points was assessed twice weekly until the wounds were healed beyond 95%. Irradiation increased wound healing time and lisinopril had no effect on this endpoint. Values are represented as means + 95% confidence intervals. * *p* < 0.05 vs. corresponding non-irradiated control.

**Figure 3:**
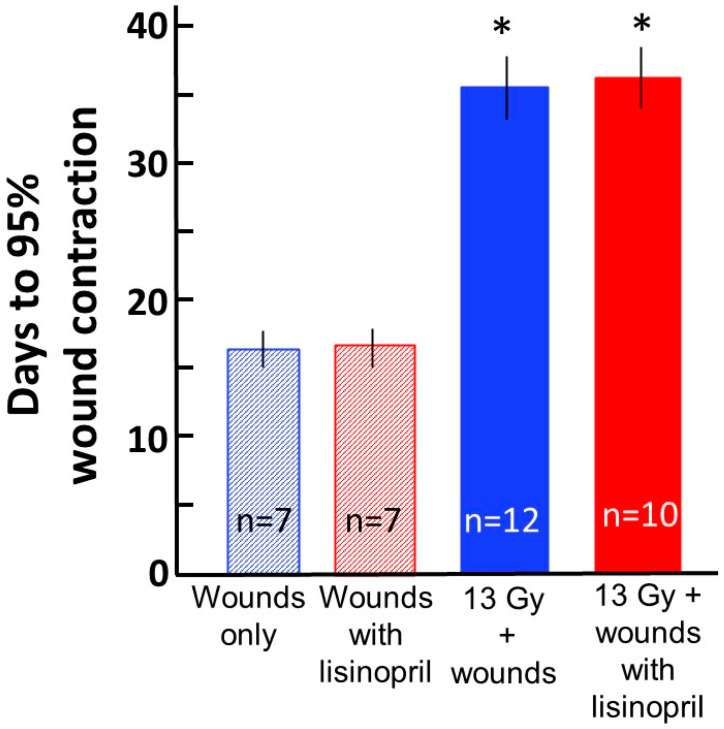
Time to wound contraction to > 95% area on day 2. Each wound area was measured at day 2 after radiation, and that area was fixed as 100%. The time in days for the wound to contract to 5% of this area (i.e., 95% contraction) is shown on the Y-axis for each group as marked below the X-axis. Non-irradiated or irradiated rats were given lisinopril starting 7 days after radiation. Values are represented as means ± 95% confidence intervals. The median time for wounds to contract to 95% after combined injury was 37 days. * *p* < 0.001 vs. the corresponding non-irradiated group. The sample size (*N*) in each group is labeled in the corresponding bar.

In a subset of wounded rats in groups (1) wounds only (*N* = 7), (2) wounds only + lisinopril (*N* = 7), (5) combined injury (*N* = 12) and (6) combined injury + lisinopril (*N* = 14), the area of each wound was measured starting at day 2 until the wound contracted to ≥ 95% of this area. The time to wound closure was measured by plotting the mean time to reach > 95% contraction in each group. The results are shown in Figure 2 and Figure 3. The wound areas on day 2 were similar in all four groups and were set at 100% (Figure 2). Wound areas decreased with time but were considerably larger in irradiated rats than the non-irradiated controls at all time points measured after day 2. As seen in Figure 2 and Figure 3, respectively, wound contraction was not altered by the ACE inhibitor lisinopril. Wound contraction to 95% was reached in 17 days in non-irradiated rats (*N* = 7/group) but was delayed to 37 days in irradiated rats (*N* = 10–12/group, Figure 3). 

### 2.3. DEARE Is Not Altered by Combined Injury but Is Improved by Lisinopril 

**Figure 4:**
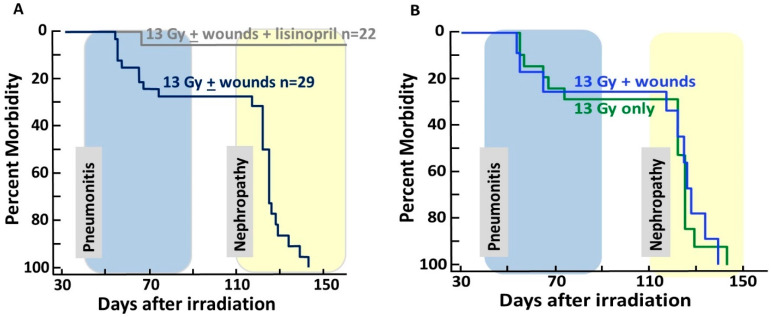
Kaplan–Meier plots depicting morbidity from 31 to 150 days after 13 Gy leg-out PBI. Following acute radiation syndrome (ARS), rats were at risk for morbidity from DEARE. Morbidity accrued secondary to radiation pneumonitis between 42 and 90 days (shaded blue) and radiation nephropathy (shaded yellow) after 110 days following 13 Gy leg-out PBI. (**A**). Irradiated and wounded rats were morbid by 150 days (navy blue line) except those receiving lisinopril (grey line) started at 7 days after radiation and continued. *p* ≤ 0.0001 between the 13 Gy leg-out PBI with or without wounds vs. 13 Gy leg-out PBI with or without wounds + lisinopril. (**B**). There is no difference in morbidity between irradiated rats with wounds (blue line) vs. irradiated only rats (without wounds, green line) if the rats were not given lisinopril.

Morbidity continued to be evaluated during DEARE from 31 days to the end of the experiment (Figure 4). Once again, rats that were not irradiated were excluded in the evaluation since there was no morbidity in these groups. There was no effect of wounds on survival during DEARE, i.e., rats with combined injuries (irradiation + wounds) had the same risk for morbidity as those given irradiation only (Figure 4A). However, there was a marked decrease in morbidity between irradiated rats given lisinopril (grey line) versus irradiated rats not given lisinopril (navy blue line, *p* < 0.0001, Figure 4A). The hazard ratio was 0.03 with 95% confidence interval of 0.004–0.262, indicating an over 30+ times decrease in morbidity by lisinopril. 

The analyses were repeated for morbidity between 31 and 90 days after radiation, which is when the rats developed pneumonitis (shaded in blue). There was no effect of wounds on survival during pneumonitis. Of the irradiated rats treated with lisinopril (with or without wounds), 1/18 became morbid as compared to 9/38 in the same groups not given lisinopril (Figure 4A), i.e., the single morbid rat given lisinopril was not wounded. Rats that did not receive lisinopril but recovered from pneumonitis succumbed to radiation nephropathy (see yellow shaded area) by 150 days (Figure 4A,B). 

### 2.4. Lisinopril Mitigates Radiation Pneumonitis after Combined Injury—Analyses by Lung Function (Secondary Endpoint)

**Figure 5:**
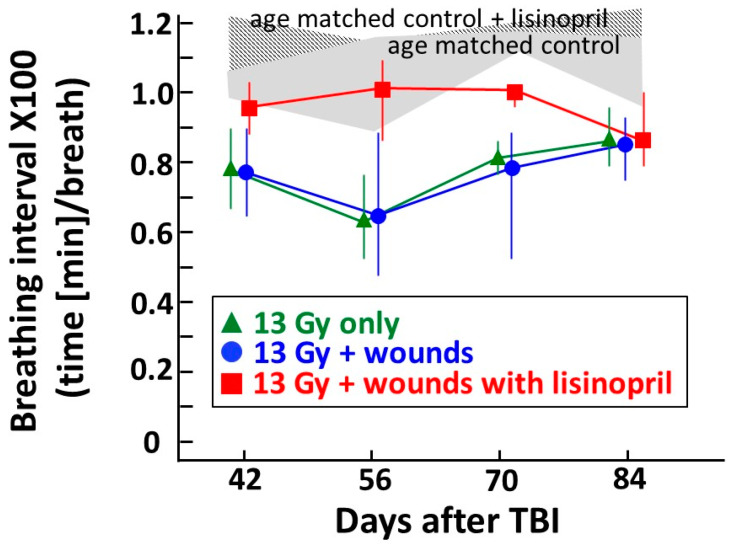
Graphical representation of breathing interval as a secondary and functional endpoint of pneumonitis, between 42 and 84 days post 13 Gy leg-out PBI with or without wound trauma. Non-irradiated, wounded rats (age matched control) or those given lisinopril (age matched control + lisinopril) have breathing intervals as shown in the shaded areas, which are similar to historical data from other studies with the same strain and sex [15]. The lines connect the median with 20–80% confidence intervals of the breathing intervals after 13 Gy leg-out PBI with or without wound trauma and also 13 Gy leg-out PBI + wounds with lisinopril started after 7 days and continued (see Materials and Methods for details). Rats morbid with lung injury confirmed at necropsy are given a breathing interval of 0 at all following time points to account for attrition. *p* ≤ 0.05 between the 13 Gy leg-out PBI+ wounds vs. 13 Gy leg-out PBI+ wounds + lisinopril groups at 42, 56 and 70 but not 84 days. Results show mitigation of radiation pneumonitis by lisinopril after irradiation only or after combined irradiation with skin wounds. *N* = 4–6/group for wounded, age-matched nonirradiated control groups; *N* = 8–12/group for irradiated groups.

Radiation pneumonitis can be quantitated by measuring breathing rates of irradiated vs. non-irradiated rats [16,17]. Data in the current study are presented as the inverse of the breathing rate, the ‘breathing interval’ to account for attrition due to morbidity during pneumonitis [18]. As the breathing rate increases, the breathing interval decreases. Rats that are morbid between 42 and 80 days are assigned a breathing interval of ‘0′ that is included in determining the median value for each following time point in the group. This allows inclusion of all rats at each time point through pneumonitis. The results are shown in Figure 5. The shaded areas represent the breathing intervals of non-irradiated rats with or without lisinopril. While the plots for the 13 Gy only or 13 Gy + wounds are almost superimposed, wounded irradiated rats that were given lisinopril (red line) had breathing intervals close to the non-irradiated rats (shaded areas). The breathing intervals of the 13 Gy only or 13 Gy + wounds decreased at 42, 56 and 70 days. Irradiated rats that survived past 80 days recovered from pneumonitis, as reflected by an increase in breathing intervals to values close to non-irradiated controls.

### 2.5. Lisinopril Mitigates Radiation Nephropathy after Combined Injury—Analyses of Kidney Function (Secondary Endpoint)

**Figure 6:**
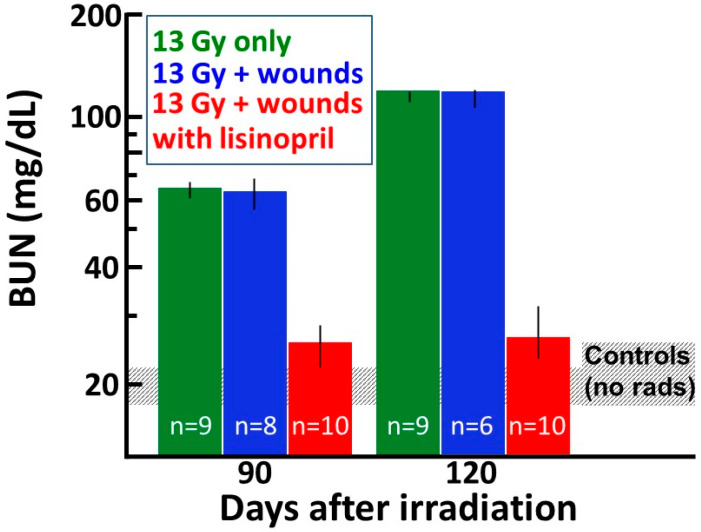
Graphical representation of nephropathy as a secondary endpoint at 90 and 120 days post 13 Gy leg-out PBI with or without wound trauma. Renal dysfunction is measured as BUN (mg/dL) shown on a log scale along the Y-axis. Non-irradiated, wounded rats only or those given lisinopril have BUN values as shown in the grey shaded bar, which are similar to historical data from other studies with the same strain and sex of age-matched non-irradiated rats [15]. The vertical bars depict the median with 20–80% percentiles of the BUN after 13 Gy leg-out PBI with or without wound trauma and also with 13 Gy leg-out PBI with wounds and lisinopril started after 7 days and continued (see Materials and Methods for details). Rats that are considered morbid with BUN > 120 mg/dL are given a value of 120 mg/dL at all time points following this measurement to account for attrition. * *p* ≤ 0.05 between 13 Gy leg-out PBI + wounds vs. 13 Gy leg-out PBI + wounds + lisinopril at 90 and 120 days. The results demonstrate mitigation of radiation nephropathy by lisinopril after combined irradiation with skin wounds. The sample size (*N*) in each group is labeled in the corresponding bar.

Radiation nephropathy leads to renal dysfunction, which can be monitored sequentially in a single cohort of rats by minimally invasive measurements of the blood urea nitrogen (BUN). To account for attrition, rats were represented by a BUN = 120 mg/dL at all time points following the one where they reached a morbid threshold (BUN of ≥120 mg/dL). The BUNs at 90 and 120 days after radiation are shown in Figure 6. Non-irradiated, wounded rats with or without lisinopril did not show elevated BUNs at the 90-day time point (BUN represented by a ‘control’ shaded bar in Figure 6). No rat had a BUN at or above the morbid threshold at 90 days after irradiation. Irradiated rats with or without wounding had a median BUN > 60 mg/dL, which was greater than triple that of the median BUN for non-irradiated rats at 90 days (see grey horizontal bar). These values were elevated at 120 days, at which time some rats had reached the 120 mg/dL BUN threshold. However, rats in the irradiated + wounds with lisinopril group still had a median value below 30 mg/dL at both time points, indicating lisinopril mitigated radiation nephropathy after combined injury.

## 3. Discussion

This study describes for the first time a combined injury model of radiation and full-thickness skin wounds that permits evaluation of both ARS and DEARE in the same animal. Wounding alone without irradiation did not impact morbidity through ARS up to 30 days. Only 3% of rats (1/30) in the irradiation alone group succumbed to morbidity between 5 and 14 days, while 15% of rats were morbid after combined injury (Figure 1). These rats were from combined injury groups with or without administration of lisinopril. Lethal ARS commenced before lisinopril was started (at 7 days post-irradiation), making it difficult to evaluate the effect of lisinopril on the ARS phase after combined injury. On the other hand, there was no significant difference in morbidity between irradiated rats with or without combined injuries during the DEARE phase, unless the combined injury groups were also treated with lisinopril. Lisinopril, which mitigates DEARE by radiation alone in the same model [15], also mitigated DEARE following combined injury, decreasing all-cause morbidity from ~70% to 0% from days 30 to 154 after irradiation. Lisinopril did not improve wound healing during ARS, even though a drug from the same family (captopril) improved this endpoint in a combined model with WTLI [12]. This dissonant finding could be attributed to the fact that, in the earlier study, only the thorax was irradiated, whereas in the PBI model in use for the current study, most of the rat, including the kidneys, was exposed. 

Since the primary endpoint was morbidity through DEARE, rats were not sacrificed at interim times for histological evaluation, which is a limitation of the study design. Additionally, histopathological measurements and comparisons were not possible at termination since all irradiated animals not treated with lisinopril were morbid. However, it has been determined that measurements of BUN are an equally sensitive indicator of radiation nephropathy in this rat model as renal histopathology [19], which is why this assay was conducted at two sequential times during DEARE. Measurements of secondary, functional endpoints consisting of breathing interval (to evaluate radiation pneumonitis) and BUN (to evaluate radiation nephropathy) confirmed lisinopril mitigated pulmonary and renal dysfunctions through DEARE. 

The results also demonstrated that, in this rat model, combined injury not only exacerbated ARS, but radiation almost doubled the time required to heal skin wounds through the ARS phase after combined injury, as compared to rats wounded but not irradiated (Figure 3). ARS is conventionally described as continuing over 30 days in rats, and though no morbidity was observed between 15 and 30 days in the current study, low circulating blood cell counts were measured at 15 days, which increased towards recovery up to 30 days in the same irradiated rat model (13 Gy leg-out PBI, results not shown). The current study also did not address the effect of wounding/wound healing, if wounds were introduced during radiation pneumonitis or nephropathy. 

The mechanisms of radiation injury and mitigation are not yet fully understood. Previous results strongly support organ-specific mechanisms of injury and recovery, since ACE inhibitors were not effective against ARS in rats [20], but they improved survival and organ function in the lung and kidney during DEARE [15]. Interestingly, captopril is reported to mitigate ARS in mice if given after but not before radiation [21,22]. The present study shows that lisinopril did not improve wound healing, but it also did not exacerbate the early radiation sequelae in rats (Figure 2 and Figure 3). Taken together, the results suggest that the renin–angiotensin system is involved in mediating radiation-induced injuries and recovery in some but not all organ systems. Other drugs have shown variable actions. For example, treatment with pegylated G-CSF, which improves ARS by enhancing recovery of circulating blood cell counts after radiation in mice, delayed wound healing after ^60^Co gamma-photon radiation followed by 15% total BSA skin wounds [5]. However, in female mice exposed to ^60^Co-γ-photon radiation at 9.5 Gy (LD_50/30_) with skin wound, ghrelin mitigated bone marrow injury and accelerated skin-wound healing by day 30 [23]. Therefore, recovery of wounds appears to be mediated by different biological pathways than recovery of the bone marrow, since the same inhibitor demonstrated variable actions on these two systems. EUK-207, a synthetic superoxide dismutase (SOD)/catalase mimetic, mitigated radiation dermatitis and enhanced skin wound healing in a combined model of radiation to the skin and overlapping full-thickness skin wounds [24], indicating injury to the skin by both radiation and wound trauma is facilitated by reactive oxygen species and free radicals. Interestingly, EUK-207 also mitigated radiation pneumonitis [25] in rats after WTLI, so oxidative injury may be common between post-radiation lung and skin sequelae. 

Other models of combined injuries have described both similar and different effects from the current rat study. Kiang et al. [5,23,26,27] reported several investigations with radiation exposure combined with skin wounds in B6D2F1/J mice. Female mice exposed to ^60^Co gamma-photon irradiation followed by 15% total BSA skin wounds experienced 25% higher mortality over 30 days compared to those given radiation alone. These findings are similar to those observed in the current study. The mouse studies, which included measurements of blood cells counts through ARS, indicated that neutrophils, lymphocytes, monocytes, eosinophils, basophils and platelets were very low at 30 days in animals surviving radiation alone, whereas only the lymphocyte counts were low in mice surviving combined injury [5]. While pegylated G-CSF increased white and red cells and platelet counts after irradiation alone, only red blood cells and platelets were increased by the drug after combined injury, indicating differential action. Similarly, treatment with the drug Alxn4100TPO (a thrombopoietin mimetic) alone increased red blood cells and platelets after irradiation but only platelets after combined injury [5]. In female mice exposed to ^60^Co-γ-photon radiation and given a skin wound, combined injury induced adipocyte counts, consistent with more injury, and fewer megakaryocytes in the sternum at day 15 than irradiation alone [23]. Skeletal tissue loss occurred with combined injury resulting in long-term (day 120) elevations in osteoclast number and sclerostin (bone formation inhibitor) [28]. In that study [28], delayed radiation-induced bone injuries were exacerbated by combined injury at day 120. In a separate study, mice were given 3 Gy of reactor-generated mixed field radiation (0.38 Gy/min, neutrons + γ-photons) followed by nonlethal skin wounding or burn [27]. Both wounds and burns reduced survival and increased C-reactive protein, complement component 3, IgM and prostaglandin E2 in serum in irradiated animals. In summary, these studies indicate more severe early and delayed damage in the combined injury groups as compared to the radiation only groups. However, differential bone-marrow damage and recovery, as well as variable effects of mitigators after radiation alone vs. radiation combined with skin trauma, make it clear that there are differences in the mechanisms of injury from radiation vs. combined injuries, which remain poorly understood.

Other investigators have focused on GI injury after radiation combined with 15% total BSA scald burn [29]. Intestinal barrier integrity was reduced by irradiation, though the largest differences were observed after combined injury at 24 and 48 h [29]. A three-fold increase in intestinal apoptosis was seen by 48 h after combined injury, and at 72 h a 100-fold increase in bacterial growth was observed in this group. In another study [30], intestinal injury persisted to 72 h in the combined injury group only. An enhanced influx of neutrophils into the GI tract contributed to a hyperinflammatory state after combined injury.

In addition to these studies in mice, rat models of radiation-induced skin injury combined with skin trauma have been reported [24,31]. Radiation injury was delivered in a single dose of a soft X-ray beam to the rat skin, without significant exposure to internal organs. Radiation exposure delayed skin wound healing, as compared to skin wounds alone in nonirradiated rats, in a dose-related manner [24]. Treatment with EUK-207 suppressed indicators of tissue oxidative stress. These results demonstrate that oxidative stress has a critical role in the progression of radiation-induced skin injuries [24].

In contrast with these results, others have demonstrated enhanced survival from acute radiation injury in mice up to one month after total body irradiation with skin wounds [9,10,11], as compared to irradiated mice that did not receive skin wounds. Survival was improved only if the skin was wounded within a narrow window of time before or after irradiation. Similar findings were reported by Garrett et al. [13], in which this group demonstrated that wounding post-irradiation reduced hematopoietic lethality in female mice. This group extended their studies to male animals demonstrating enhanced survival by combined injuries in both sexes. Between 6.2 and 6.7 Gy, the increase in survival was associated with enhanced recovery of hematopoiesis, preceded by an increase in select cytokines. As already mentioned, radiation to the skin given 3 h after whole thorax irradiation mitigated lung morbidity (radiation pneumonitis) [12]. The same papers demonstrated that the ACE inhibitor captopril accelerated the rate of wound healing in this combined injury model, though no improvement in wound healing was observed if lisinopril was given after leg-out PBI with wounds in the current study (Figure 2 and Figure 3). 

The leg-out PBI model involved exposure of the total body to irradiation except for part of one hind leg, shielding ~8% bone marrow [15]. Early supportive care with fluids and antibiotics was given to mitigate GI toxicity. This unique rat model appears to be only one that follows irradiated subjects until they are all morbid, and involves irradiation to every organ except a minimal portion of the bone marrow to support repopulation for survival through the ARS phase. 

The goal of this study is to develop a terrorism-relevant injury model by combining leg-out PBI with skin trauma. Male rats were not selected since the research team had a well-characterized leg-out PBI injury model available for females [15]. Further studies must be conducted in male rats and pediatric and geriatric rats since differences in injury and mitigation have been reported in these special populations [32,33]. More studies are also needed to determine the mechanisms of radiation injury and mitigation of DEARE by lisinopril, after irradiation alone or with combined injuries. 

The growing risks of combined injuries from nuclear accidents, in the context of ongoing global tensions, also emphasizes the need for future studies in this field. Several countries have developed nuclear weapons increasing the chances of radiological attacks by terrorists. Data derived from the atomic events in Hiroshima, Nagasaki and Chernobyl suggest that > 40% of injuries observed were from radiation exposure combined with other insults, such as thermal burns or skin lacerations [7,8,34]. In addition, infection could complicate the outcomes [6]. The sequelae of organ injuries in the rat model of single or combined injuries are similar to those observed in humans and nonhuman primates [1]. In addition, since the scenarios that are being simulated involve mass casualty events to large populations, pharmacological or other interventions cannot be given before radiation but must be started after exposure (preferably at least 24 h post-irradiation, which is a minimal window of time for dosimetry, triage and treatment). For this reason, the mitigator lisinopril was started 7 days after radiation in the current study. Even after this delayed start, the drug was effective in mitigating pulmonary and renal dysfunction during DEARE. 

## 4. Conclusions

Combined injuries will occur from a radiological attack or nuclear accident. The rat leg-out PBI model which includes irradiation of the whole body, except for ~8% of bone marrow in one hind limb, can be used to study the effects of combined injury with whole-thickness skin wounds. Skin wounds given immediately after irradiation increase morbidity from the ARS phase in this model, while irradiation delays wound healing within the first 38 days. Skin wounds with irradiation do not exacerbate radiation pneumonitis or nephropathy, which occur after the wounds have healed. The ACE inhibitor lisinopril started 7 days after leg-out PBI mitigates the delayed effects of radiation in wounded and non-wounded rats but does not enhance or accelerate wound healing. Lisinopril could not be conclusively evaluated for effects on ARS in the schedule given. The results from this study suggest different molecular mechanisms of injury and healing during the ARS and DEARE.

## 5. Materials and Methods

### 5.1. Animal Care

The animal protocols (AUA0000246(260-05-1)) and identification of morbid rats for euthanasia were approved on 06/13/2019 by the Institutional Animal Care and Use Committees (IACUC) at the Medical College of Wisconsin, Milwaukee (D16-00064(A3102-01)). 

### 5.2. Groups

A total of six groups of rats included (1) skin wounds only (*N* = 7), (2) wounds with lisinopril (*N* = 7), (3) 13 Gy leg-out PBI only (*N* = 22), (4) 13 Gy leg-out PBI with lisinopril (*N* = 8), (5) 13 Gy leg-out PBI and wounds (combined injury) (*N* = 16) and (6) combined injury with lisinopril (*N* = 14). The number of rats in each group was adjusted based on studies with similar treatments [12,15,24]. 

### 5.3. Animals and Irradiation

*Leg–out partial body irradiation (PBI) in rats:* This protocol was the same as that reported in previous studies [15]. Dosimetry was as described by Medhora et al. [34]. Briefly, WAG/RijCmcr female rats were immobilized in a plexiglass jig and irradiated without the use of anesthetics at 11 to 12 weeks of age (~155 g). One hind limb of each rat was carefully shielded with a 0.25 inch lead block. An XRAD 320KV orthovoltage X-ray system (Precision X-Ray, North Branford, CT, USA.) was operated at 320 kVp and 13 mAs with a half value layer of 1.4 mm Cu with a dose rate of 1.75 Gy min^−1^ for a total dose of 13 Gy. The dose to the shielded hind limb was measured to be ~2 Gy for a 13 Gy leg-out PBI exposure [15]. Non-irradiated rats were sham irradiated.

### 5.4. Supportive Care and Treatment with Lisinopril

All rats were given supportive care consisting of enrofloxacin (10 mg kg^−1^day^−1^) in the drinking water from days 2 to 14 post-irradiation, and hydration by daily subcutaneous injection of saline (40 mL kg^−1^day^−1^) corresponding to days 3 to 7 post-irradiation. Rats in groups 2, 4 and 6 were also given lisinopril (21CEC PX Pharm Ltd., Sussex, UK; 40 mg/L in the drinking water (vehicle) for an approximate dose of 24 mg.m^2^.day^−1^). The water was changed weekly through which time the drug was stable in solution. Lisinopril was started 7 days after the day of radiation and continued until the experiment was terminated.

### 5.5. Wounding

Rats received two circular, full-thickness skin wounds on their back below the neck, within 2 h after irradiation as described previously [24]. Each wound was 8 mm in diameter. The analgesic carprofen (Pfizer, New York, NY, USA.) was injected at 5 mg/kg subcutaneously one hour prior to wounding. Rats that were irradiated but not wounded were also shaved and given analgesic. Wounds were left uncovered, and the animals housed singly according to IACUC protocol until the wounds healed. Rats were monitored very carefully for pain or distress, especially during the first 48 h after wounding.

### 5.6. Measurement of Skin Wounds 

Wound contraction, an integral part of wound healing [24], was assessed twice weekly until the wounds healed. Wounds were traced on clear autoclaved plastic, and the images were scanned and used to calculate injured surface area as described [24]. To determine wound contraction twice weekly, the area of the two wounds on each rat was averaged, calculated and normalized to the day 2 averaged area for that rat. The normalized averages were used for statistical analysis. The time to > 95% contraction was determined to measure the time to wound healing in each experimental group. 

### 5.7. Measurement of Breathing Interval

As a quantitation of radiation pneumonitis, breathing rates for each rat were measured every two weeks from days 42 to 84 post-irradiation as described previously [18,35]. Briefly, rats were restrained in a plethysmograph (Scireq, Emka Technologies Co. Montreal, Canada). The frequency of pressure changes that were mainly due to respiratory movement was recorded and analyzed. Recordings for a maximum of 10 min per animal were used. The mean breathing rate for each rat was then calculated from four steady regions of the recording lasting greater than 15 s each. The inverse of the breathing rates was calculated to derive the breathing interval or time/breath in minutes. Since a higher breathing rate and lower breathing intervals are associated with more lung damage, the breathing interval was set to 0 min for all animals that died during pneumonitis to account for attrition [18]. 

### 5.8. Measurement of Blood Urea Nitrogen (BUN) 

Previous published work has shown that rising BUN levels correlate well with histopathological damage and are a sensitive, functional and minimally invasive method to assess radiation nephropathy [36]. Rats were anesthetized by inhalation of 3–5% isoflurane, 0.3–0.5 mL of blood was harvested and serum was collected and frozen for the BUN colorimetric assay, as described previously [35,37]. BUN was expressed as mg/dL of serum, and medians with 20–80% ranges were used for statistical analysis. Irradiated rats with BUN ≥120 mg/dL were euthanized and given a value of 120 mg/dL to account for attrition, since such rats were previously confirmed to have severe and irreversible lethal renal damage [38].

### 5.9. Statistical Analyses

#### 5.9.1. Morbidity

Rats receiving sham irradiation (groups 1 and 2) were excluded from these statistics since no rats were morbid at termination of the experiment. 

Analysis for 30-day morbidity (ARS): The associations between wounds and morbidity, and between lisinopril and morbidity for all irradiated rats, were examined by Fisher’s exact tests as no censoring was observed within 30 days in these groups. The interaction effect of wounds and lisinopril on 30-day morbidity was not estimable due to insufficient number of events in the lisinopril group. 

Analysis for morbidity after 30 days (DEARE): The interaction between wounds and lisinopril on time to morbidity for irradiated rats could not be examined due to the absence of morbidity in the group of rats with lisinopril and without wounds. Cox proportional hazard models were used to determine if wounds or lisinopril were associated with time to morbidity, and the validity of the proportional hazards assumption was confirmed with a Cox proportional hazard test. The effect of lisinopril on survival between 31 and 90 days (during pneumonitis) was also evaluated using Log Rank tests. This analysis was validated by Fisher’s exact test.

#### 5.9.2. Statistics for Functional, Secondary Endpoints

Wound sizes were expressed as means ± 95% confidence intervals. Comparisons for effect of radiation or lisinopril on wound healing were made separately for irradiated (13 Gy) or non-irradiated (0 Gy) groups (0 Gy vs. 13 Gy) and with or without lisinopril (0 Gy vs. 0 Gy + lisinopril, 13 Gy vs. 13 Gy + lisinopril) by separate t-tests at each time point. Similar independent t-tests were also used to compare the time to reach > 95% wound contraction either with radiation or with lisinopril. Breathing intervals and BUN values are shown as medians and 20%–80% ranges. Statistical differences in breathing intervals at each time point were compared with Kruskal–Wallis ANOVA on ranks, with adjustments for multiple comparisons vs. the control group by Dunnett’s method. Statistical pairwise differences between groups for BUN were calculated by the Mann–Whitney U test. Both the breathing interval and BUN analysis accounted for attrition as described in the text.
